# Editorial: Iatrogenic neurotoxicity – Mechanisms, prevention, and treatment

**DOI:** 10.3389/fnins.2023.1184317

**Published:** 2023-03-22

**Authors:** Dragica Selakovic, Daoud Ali, Aziz Eftekhari, Nemanja Jovicic, Gvozden Luka Rosic

**Affiliations:** ^1^Department of Physiology, Faculty of Medical Sciences, University of Kragujevac, Kragujevac, Serbia; ^2^Department of Zoology, College of Science, King Saud University Riyadh, Riyadh, Saudi Arabia; ^3^Department of Biochemistry, Faculty of Science, Ege University, Izmir, Türkiye; ^4^Department of Histology and Embryology, Faculty of Medical Sciences, University of Kragujevac, Kragujevac, Serbia

**Keywords:** neurotoxicity, therapy adverse effects, iatrogenic, prevention, treatment

The aim of the Research Topic “*Iatrogenic neurotoxicity – Mechanisms, prevention, and treatment*” was to provide an integrated view of the state-of-the-art research on the basic mechanisms underlying iatrogenic neurotoxicity, as well as by providing a comprehensive insight of the topic through original research and review articles focusing on iatrogenic neurotoxicity. Although there is general awareness of the fact that the neurotoxicity is one of the most frequent adverse effects in various therapeutic protocols, the review of literature data confirmed that other manifestations of systemic toxicities, such as hepato- and nephro-toxicity, get more attention while the varieties of manifestations of neurotoxicity are often concealed, and consequently untreated. Furthermore, some manifestations of iatrogenic neurotoxicity are still misdeclared and attributed to social and environmental outcomes of surrounding artifacts accompaning specific therapeutic protocols.

Herewith, we provide a brief Editors' presentation of the articles published in this Research Topic.

Following the well-known and etiologically confirmed dyskinesia accompanied by the standard employment of levodopa in neurodegenerative diseases therapy, Wan et al. chose an optimal animal model (6-OHDA lesioned striatum of LID rat model) for the estimation of the signaling pathway underlying neuroinflammation, as the principle pathophysiological background of this iatrogenic adverse effect. Indeed, the applied methodology confirmed L-dopa-induced overexpression of the pro-inflammatory mediators and overactivation of the canonical NF-κB signaling pathway, but this was attenuated by the inhibition of BET protein function using JQ1, suggesting its protective role *via* suppression of the canonical NF-κB signaling pathway.

The neuroinflammation has also been considered as the key cause of cognitive impairment induced by anesthesia (especially prolonged), which is generally considered to be the key cause of cognitive impairment that may result in long-lasting cognitive decline in vulnerable categories, such as neonatal subjects. Zhang et al. confirmed that effect of isoflurane on neonatal mice in behavioral testing followed by biochemical and morphological analyses. However, they also reported that pretreatment with disodium cromoglycate attenuated the impact of anesthesia on cognitive function by reducing mast cells degranulation and mast cell tryptase expression. The presented protocol was sufficient to attenuate neuroinflammation, activation of microglia and astrocytes, and damage to oligodendrocytes and synapses underlying cognitive impairment.

In contrast to the studies on neonatal mice, the investigation of Borgstedt et al. showed no significant impact of isoflurane on cognitive and/or behavioral performance in a mouse model of early-stage Alzheimer's disease performed on adult mice (10 weeks). Thus, typical cognitive impairment (accompanied with amyloidopathy, inflammation, and apoptosis) was not worsened by general anesthesia, still remaining significantly above the values observed in wild type animals. Also, it is worth noting that according to the results of this study, neurodegeneration and clinical symptoms showed no gender differences in this animal model, in accordance with how it has been described in human population.

A different methodological approach was employed by Chen et al. for the estimation of neurotoxic effects of another general anesthesia drug. An *in vitro* study performed on cell culture (on neurons present in the hippocampus, brain region involved in cognitive control), showed that propofol induced numerous adverse effects, including decreased neuronal viability, mitochondrial membrane alterations, increased indicators of oxidative damage, apoptosis, and activation of ferroptosis. Although there was no ethological connection, the authors experimentally confirmed that hypoxic preconditioning was sufficient to reduce the neurotoxicity of propofol by inhibiting ferroptosis.

In contrast to the previously discussed original research articles, this Research Topic was concluded by a very useful overview considering the nanoparticles-induced neurotoxicity by Zia et al.. Although with different origins, routes of administration, and a variety of indications, nanoparticles-induced neurotoxicity usually depends on their ability to bypass the blood-brain barrier. The most frequently described pathway for nanoparticles to enter the central nervous system is *via* sensory neurons and receptors, resulting in oxidative damage, disturbance in immune response, and genome dysfunction. In their review, the authors also discussed the cope-up strategies to overcome nanoparticles-induced neurotoxicity that include plant-based antioxidants, essential oils, and dietary supplements.

Taken altogether, iatrogenic neurotoxicity still remains insufficiently evaluated, but clinically extremely important outcome of numerous currently used medical procedures. Therefore, any effort in the estimation of its mechanisms and potential cope-up strategies to overcome its frequency and severity may significantly reduce the problems accompaning this spectrum of adverse effects.

## Author contributions

All authors listed have made a substantial, direct, and intellectual contribution to the work and approved it for publication.

